# Effect of Intravenous Magnesium on Postoperative Pain and Analgesic Outcomes in Patients Undergoing General Anesthesia: A Systematic Review and Meta-Analysis of Randomized Controlled Trials

**DOI:** 10.3390/ph19081296

**Published:** 2026-08-16

**Authors:** Lucyna Tomaszek, Wioletta Mędrzycka-Dąbrowska, Sandra Lange, Sebastian Dąbrowski, Mateusz Szczupak

**Affiliations:** 1 Faculty of Health Sciences, Andrzej Frycz Modrzewski Krakow University, 30-705 Kraków, Poland; ltomaszek@uafm.edu.pl; 2 Department of Thoracic Surgery, Institute of Tuberculosis and Lung Diseases, 34-700 Rabka-Zdrój, Poland; 3 Department of Anaesthesiology Nursing & Intensive Care, Faculty of Health Sciences, Medical University of Gdańsk, Dębinki 7, 80-211 Gdańsk, Poland; 4 Department of Anesthesiology and Intensive Care, University Clinical Center, Dębinki 7, 80-952 Gdańsk, Poland; 5 Department of Internal and Pediatric Nursing, Medical University of Gdańsk, Dębinki 7, 80-211 Gdańsk, Poland; 6 Department of Medical Rescue, Faculty of Health Sciences, Medical University of Gdańsk, Dębinki 7, 80-211 Gdańsk, Poland; 7 Department of Anaesthesiology and Intensive Therapy, Nicolaus Copernicus Hospital in Gdańsk, 80-808 Gdańsk, Poland; 8 Department of Otolaryngology, Faculty of Medicine, Medical University of Gdańsk, 80-210 Gdańsk, Poland

**Keywords:** postoperative pain, magnesium sulfate, intravenous magnesium, multimodal analgesia, opioid consumption, opioid-sparing, randomized controlled trials, meta-analysis, general anesthesia, perioperative analgesia

## Abstract

**Background:** Intravenous magnesium sulfate has been proposed as an adjuvant analgesic in perioperative care; however, its effects on postoperative pain and related analgesic outcomes remain uncertain. **Methods:** A systematic search of the literature was conducted from 1 January 2015 through 19 January 2026 to identify randomized controlled trials comparing intravenous magnesium sulfate with placebo in adults undergoing general anesthesia. Pain intensity was harmonized to a 0–10 scale. Primary outcomes were early postoperative pain (0–6 h), pain at 12 h, and pain at 24 h. Secondary outcomes were postoperative opioid consumption, expressed as intravenous morphine equivalents, and time to first rescue analgesia. Random-effects meta-analyses were performed. Risk of bias was assessed using RoB 2, and certainty of evidence using GRADE. **Results:** A total of 24 randomized controlled trials were included in the qualitative synthesis, of which 22 provided data for quantitative meta-analysis, yielding 23 comparisons and a total of 1750 participants. Intravenous magnesium reduced early postoperative pain (mean difference [MD] −0.90, 95% confidence interval [CI] −1.33 to −0.47; I^2^ = 96.27%) and pain at 24 h (MD −0.59, 95% CI −1.00 to −0.18; I^2^ = 66.36%), but not pain at 12 h (MD −0.52, 95% CI −1.15 to 0.11; I^2^ = 77.38%). Magnesium also reduced postoperative opioid consumption (MD −6.28 mg morphine equivalents, 95% CI −8.94 to −3.61; I^2^ = 95.93%) and prolonged time to first rescue analgesia (MD 85.61 min, 95% CI 40.11 to 131.10; I^2^ = 79.37%). Certainty of evidence ranged from very low to moderate, with the highest certainty observed for pain at 24 h. **Conclusions:** Intravenous magnesium was associated with reduced postoperative pain, particularly at 24 h, and with opioid-sparing effects. However, substantial heterogeneity across outcomes and generally low-to-moderate certainty of evidence warrant cautious interpretation. Magnesium may be considered as part of multimodal analgesia in appropriately selected patients.

## 1. Introduction

Postoperative pain remains a major challenge in perioperative care, affecting a substantial proportion of patients undergoing surgery under general anesthesia. Despite broad implementation of multimodal analgesia, inadequate pain control persists and is associated with delayed recovery, increased opioid consumption, and a higher risk of chronic postsurgical pain [1,2,3]. Opioids still play a central role in postoperative analgesia; however, their use is limited by dose-dependent adverse effects, including respiratory depression, nausea, vomiting, ileus, and risk of dependence [4,5]. These limitations have driven continuing efforts to develop effective opioid-sparing strategies within enhanced recovery pathways [6], making magnesium sulfate one of the most intensively investigated non-opioid adjuncts in this context [7,8].

### 1.1. Background

Magnesium sulfate (MgSO_4_) has become a subject of growing interest as a perioperative analgesic adjuvant. Its proposed mechanisms include antagonism of N-methyl-D-aspartate (NMDA) receptors and modulation of calcium influx, both of which are implicated in central sensitization and nociceptive transmission [9,10]. Increasing extracellular magnesium concentrations may additionally modulate the perioperative inflammatory response by reducing the release of inflammatory cytokines, thereby affecting both the intensity and duration of pain [11,12]. Through these mechanisms, magnesium may enhance analgesic efficacy and reduce opioid requirements [13]. In addition, it is inexpensive, widely available, and generally well tolerated when administered intravenously under appropriate monitoring [14]. Nevertheless, magnesium may prolong neuromuscular blockade and sedation, thus delaying recovery, and, at higher plasma concentrations, contribute to bradyarrhythmia and negative inotropy; in some trials, these effects were considered to outweigh the analgesic benefit [15].

### 1.2. Existing Evidence and Remaining Uncertainty

Since the first randomized trial of intravenous magnesium as an analgesic adjuvant in 1996 [16], subsequent randomized controlled trials (RCTs) have evaluated intravenous magnesium across a wide range of surgical settings; however, reported effects on postoperative pain and analgesic outcomes have been inconsistent [17,18]. This is not readily explained by a single factor but rather likely reflects heterogeneity in study populations, surgical procedures, dosing regimens, or timing of administration. Previous meta-analyses have suggested a potential analgesic and opioid-sparing effect [19,20], but many included older trials that might not reflect contemporary anesthetic practice and modern multimodal analgesia protocols. De Oliveira et al. pooled 20 RCTs (1257 patients) restricted to intravenous administration under general anesthesia and reported a benefit for pain at rest at ≤4 h and at 24 h, together with a substantial opioid-sparing effect; heterogeneity was high across these analyses, no effect was demonstrated on time to first analgesic request and no dose–response relationship was identified [12]. A more recent synthesis of 51 trials in non-cardiac surgery confirmed a modest reduction in 24 h morphine consumption while finding no significant effect on 24 h pain scores [21].

This updated synthesis is justified by some limitations of the prior evidence. The pooled estimates are dominated by trials conducted between 1996 and 2012, before enhanced recovery protocols, protocolized non-opioid analgesia, and regional techniques became common components of perioperative care [6,18]. Several reviews pooled across routes of administration (intravenous, intrathecal, epidural, and intra-articular), across general and regional anesthesia, and in some cases across adult and pediatric populations. Few adequately powered studies have investigated dose and timing as sources of heterogeneity, and the available evidence is inconsistent—one review found greater effects when magnesium was continued into the postoperative period, yet no relationship with cumulative dose [12]. Because previous syntheses have generally limited their reporting to confidence intervals, rarely supplementing these with prediction intervals, and have varied in whether outcome-level risk of bias (RoB 2) and GRADE were formally assessed, the plausible range of effects in a novel perioperative setting and the certainty of evidence for each outcome remain poorly defined.

Consequently, although magnesium is readily accessible and theoretically plausible as an analgesic adjunct, it still remains uncertain whether intravenous magnesium produces a clinically meaningful reduction in postoperative pain at defined postoperative time points in contemporary practice, and whether any such effect is accompanied by consistent opioid-sparing. To address this gap, we conducted a systematic review and meta-analysis of randomized controlled trials published between 2015 and March 2026 to evaluate the effect of intravenous magnesium sulfate on postoperative pain intensity in adult patients undergoing general anesthesia. The primary outcomes were pain intensity at predefined postoperative time points, including early pain (0–6 h), 12 h, and 24 h. Secondary outcomes included postoperative opioid consumption and time to first rescue analgesia.

## 2. Methods

### 2.1. Study Design

This systematic review and meta-analysis of randomized controlled trials was conducted in accordance with the PRISMA guidelines [22]. The protocol was prospectively registered in the PROSPERO registration no. CRD420261284020 and remains publicly accessible at https://www.crd.york.ac.uk/PROSPERO/view/CRD420261284020/, accessed on 20 January 2026.

### 2.2. Search Strategy

The literature search was conducted by two reviewers (WMD and SL). Four electronic databases—Scopus, PubMed, Web of Science, and CINAHL—were systematically searched from 1 January 2015 to 19 March 2026 to identify eligible randomized controlled trials. Prior to the finalization of the manuscript, the search was updated in March 2026 using the same search strategy and eligibility criteria to identify any additional eligible studies published after the initial search. The search strategy combined terms related to magnesium (e.g., magnesium, magnesium sulfate, magnesium supplementation, magnesium infusion) and postoperative pain (e.g., postoperative pain, pain, pain intensity), along with terms for randomized controlled trials. The reference lists of the studies retrieved in the initial search were screened for further eligible publications. Only studies published in English were considered. Full search strategies for each database are provided in Supplementary Table S1.

### 2.3. Eligibility Criteria

Randomized controlled trials were eligible if they included adult patients undergoing surgery under general anesthesia and compared intravenous magnesium sulfate—administered as a bolus, continuous infusion, a combination of pre-induction and intraoperative administration, or intraoperative administration alone—with placebo (saline). To be included, studies had to report at least one prespecified postoperative analgesic outcome, including early postoperative pain (0–6 h), pain at 12 h, pain at 24 h, postoperative opioid consumption, or time to first rescue analgesia, using validated pain scales such as the Visual Analogue Scale (VAS), Numeric Rating Scale (NRS), Verbal Numeric Rating Scale (VNRS), or Numeric Pain Rating Scale (NPRS), and to provide sufficient numerical data to enable effect size calculation.

Exclusion criteria were non-intravenous magnesium administration, non-randomized study designs, publications available only as conference abstracts, reviews, or meta-analyses, and studies lacking a separate magnesium study arm.

### 2.4. Study Selection

Two reviewers (WMD and SL) independently screened titles and abstracts identified through the search. Full-text articles were subsequently assessed for eligibility. Disagreements were resolved by consensus, with a third reviewer (LT) consulted where necessary. Reference lists of the included studies were screened manually for further relevant research.

### 2.5. Data Extraction

Two reviewers (LT and WMD) independently performed data extraction using a standardized protocol. Extracted data included first author, year of publication, sample size, participant characteristics (age and sex), details of the magnesium intervention, postoperative analgesic regimens and routes of administration, and trial registration identifiers where available. When required data were missing or incompletely reported, attempts were made to contact the corresponding authors to obtain additional information. Studies for which sufficient data could not be obtained were excluded from the quantitative analysis. When pain was assessed both at rest and on movement, only the resting scores were used. Numerical data reported only graphically were extracted using WebPlotDigitizer (version 5) and were included exclusively in sensitivity analyses. Discrepancies were resolved through discussion with a third reviewer (SL).

### 2.6. Risk of Bias Assessment and Evidence Grading

Risk of bias was assessed using the Cochrane Risk of Bias 2 (RoB 2) tool across the following domains: bias arising from the randomization process; deviations from intended interventions; missing outcome data; measurement of the outcome; and selection of the reported result. Each domain was judged as low risk, some concerns, or high risk according to the RoB 2 algorithm [23].

The assessment was performed at the outcome level. For studies reporting postoperative pain, risk of bias was evaluated separately for early pain (0–6 h), pain at 12 h, and pain at 24 h. For studies reporting only secondary outcomes (e.g., opioid consumption or time to rescue analgesia), the assessment was conducted for the respective outcomes. For presentation purposes, a single risk-of-bias judgment per study was assigned based on the outcome contributing to the primary analysis, or otherwise on the outcome included in the meta-analysis. The overall risk-of-bias judgement for each study corresponded to the highest level of risk recorded across domains.

All included studies were assessed irrespective of their inclusion in the quantitative synthesis. Risk of bias assessment was performed independently by three reviewers (L.T, W.M-D, S.L), with disagreements resolved by consensus or consultation with a fourth reviewer. Studies at high risk of bias were not excluded a priori but were explored in sensitivity analyses.

The certainty of evidence was assessed using the GRADE framework separately for each outcome, based on the primary analysis [24].

### 2.7. Data Processing and Standardization

Pain scores were harmonized to a 0–10 scale (0–100 scales divided by 10; original 0–10 scales used directly). Time to rescue analgesia was standardized to minutes by converting hours to minutes (×60).

Opioid doses were converted to intravenous morphine equivalents (IME) using ESAIC ENCORE conversion tables (morphine IV: 1; pethidine IV: 0.13; fentanyl IV: 0.066) [25].

When opioid consumption was reported in mg/kg as in Ghaffaripour et al. [26], total dose was estimated assuming a standard body weight of 70 kg if study-specific values were unavailable. For outcomes reported across multiple postoperative intervals, mean values were summed to obtain 24 h totals, and standard deviations were combined assuming independence of intervals.

When outcomes were reported as medians with ranges or interquartile ranges, mean and standard deviation were estimated using established methods [27,28]. Transformation was applied only to data considered approximately symmetric; clearly skewed or insufficient data were not transformed and were excluded from the primary quantitative synthesis but retained for qualitative assessment, in line with recommendations to avoid inappropriate assumptions of normality [29]. An online calculator was used to support these transformations (https://www.math.hkbu.edu.hk/~tongt/papers/median2mean.html) (accessed on 10 April 2026).

### 2.8. Statistical Data Analysis

Meta-analyses and meta-regressions were performed using STATISTICA (StatSoft Polska, Kraków, Poland, Zestaw Plus v5.1.0, 2026). All statistical tests were two-sided, and *p* < 0.05 was considered significant.

For continuous outcomes, mean difference (MD) on the 0–10 scale was calculated. Random-effects models (DerSimonian-Laird) were applied.

For trials with multiple intervention arms, each comparison was included separately, and shared control groups were accounted for to avoid double-counting.

Heterogeneity was assessed using I^2^, τ^2^, and Cochran’s Q test, and interpreted according to Cochrane Handbook guidance (I^2^ values of 0–40% might not be important, 30–60% may represent moderate heterogeneity, 50–90% substantial heterogeneity, and 75–100% considerable heterogeneity). In addition, 95% prediction intervals were calculated to estimate the expected range of treatment effects in future clinical settings, as recommended for random-effects meta-analyses [30]. Prediction intervals were calculated using established methods for random-effects meta-analysis [31].

Prespecified subgroup analyses were conducted according to timing of administration (pre-induction plus intraoperative vs. intraoperative only), surgical category (spine vs. non-spine), dosing strategy (>30 vs. ≤30 mg·kg^−1^), publication period (≤2020 vs. >2020), and trial registration status. Subgroup effects were formally assessed using random-effects meta-regression models with binary moderators coded as indicator variables (0/1).

Random-effects meta-regression analyses were performed to evaluate the association between treatment effect and continuous study-level moderators, including magnesium loading dose (mg·kg^−1^), mean patient age, and proportion of female participants. These analyses were considered exploratory.

The primary analysis included studies with low risk of bias or some concerns that reported numerical data directly, as well as studies with reconstructed mean and standard deviation values derived from non-parametric summaries, provided the data were considered approximately symmetric.

Sensitivity analyses were performed to assess the robustness of the findings and additionally incorporated studies at high risk of bias and those with graphically extracted data.

### 2.9. Publication Bias

Publication bias was assessed for outcomes with at least 10 studies. Funnel plots were visually inspected, and both Egger’s regression test and Begg’s rank correlation test were used to assess funnel plot asymmetry and possible small-study effects [32].

## 3. Results

### 3.1. Study Selection

**Figure 1:**
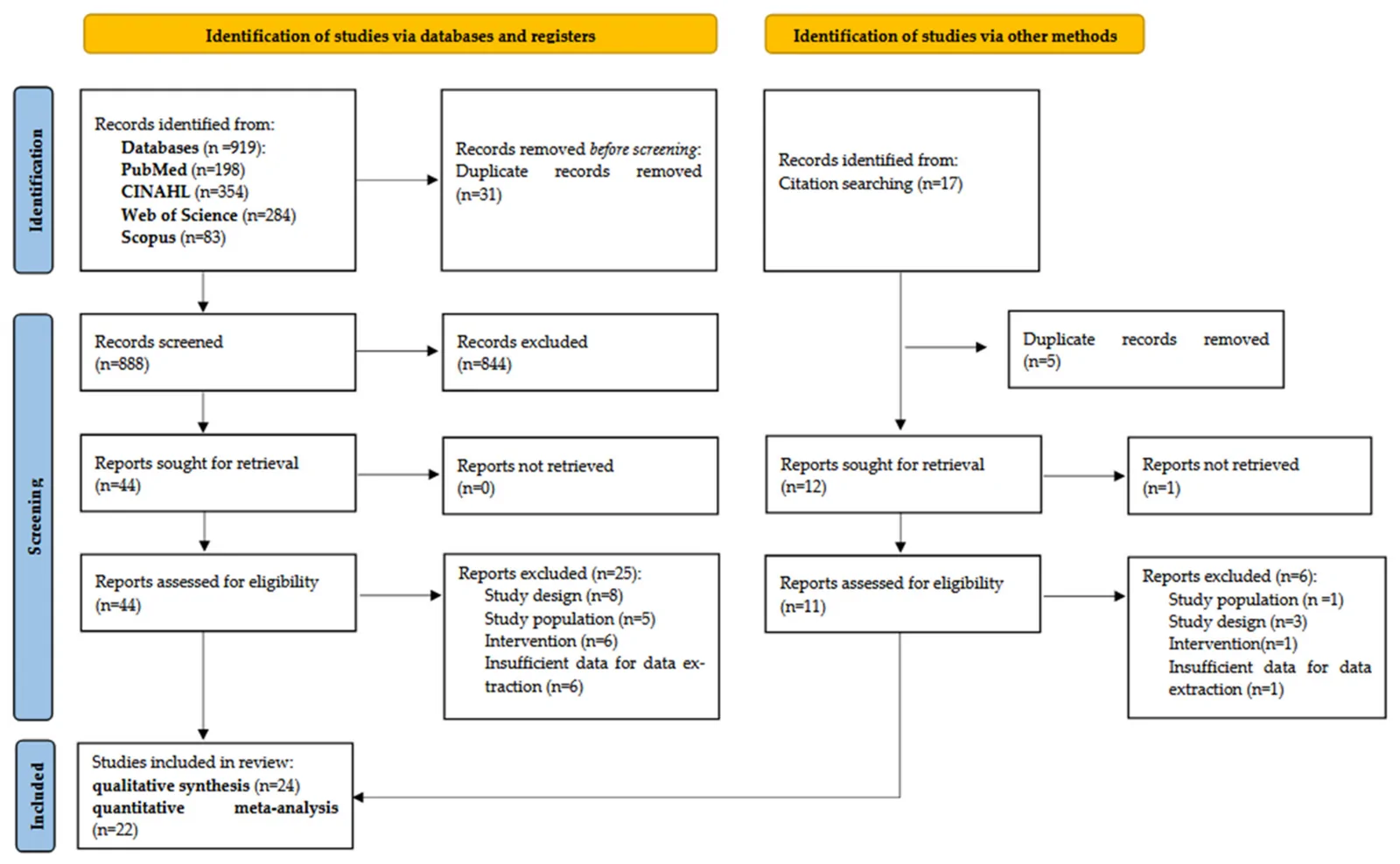
PRISMA flow diagram.

A total of 919 records were initially retrieved from the four databases. After removing duplicates (31) and screening titles and abstracts, 844 records were excluded. A total of 44 full-text studies were assessed for eligibility. The primary reasons for exclusion were: study design, wrong population/intervention and insufficient data for data extraction. An additional 17 records were identified by checking references. Of these 17 records, five were duplicates and were removed before the full-text retrieval stage. As a result, 12 reports were selected for full-text evaluation. Of these, 11 reports were assessed for eligibility. Ultimately, 24 randomized controlled trials were included in the qualitative synthesis, of which 22 provided data for quantitative meta-analysis Figure 1 [26,33,34,35,36,37,38,39,40,41,42,43,44,45,46,47,48,49,50,51,52,53,54,55].

### 3.2. Risk of Bias in Studies

**Figure 2:**
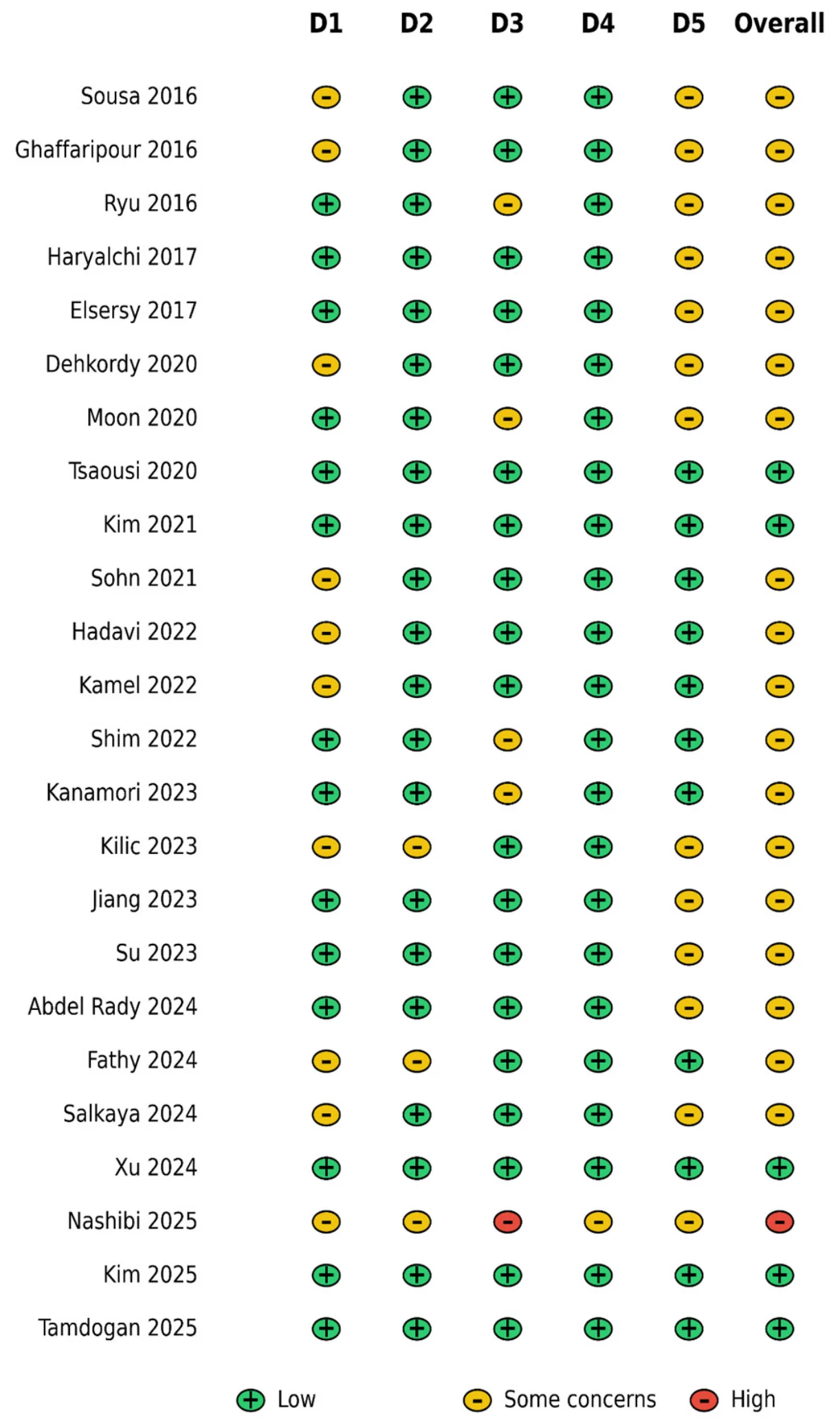
Risk-of-bias assessment. Traffic-light plot of the risk-of-bias assessment for all included randomized controlled trials using the Cochrane Risk of Bias 2 (RoB 2) tool. Domains: D1, bias arising from the randomization process; D2, bias due to deviations from intended interventions; D3, bias due to missing outcome data; D4, bias in measurement of the outcome; and D5, bias in selection of the reported result. Green, yellow, and red circles indicate low risk of bias, some concerns, and high risk of bias, respectively. The overall risk of bias was determined according to the highest level of risk across domains [26,33,34,35,36,37,38,39,40,41,42,43,44,45,46,47,48,49,50,51,52,53,54,55].

The methodological quality of all included randomized controlled trials was assessed using the Cochrane Risk of Bias 2 (RoB 2) tool (Figure 2, Supplementary Figure S1). Overall, the risk of bias was judged as low in 5 studies (20.83%), some concerns in 18 studies (75.00%), and high in 1 study (4.17%). Some concerns were most frequently identified in the randomization process (D1) and in the selection of the reported result (D5). A high risk of bias was identified in one study [54], primarily driven by substantial missing outcome data (D3).

### 3.3. Characteristics of Included Studies

A total of 24 randomized controlled trials were included in the qualitative synthesis (Table 1). Of these, 22 trials provided data suitable for quantitative synthesis. One study reported two independent magnesium intervention arms, which were treated as separate comparisons [54]. Accordingly, 23 comparisons comprising 1750 participants were included in the meta-analysis.

Two studies [45,48] were excluded from the primary quantitative meta-analysis because their outcome data were clearly skewed and could not be reliably transformed into mean and standard deviation values. Nevertheless, these studies were retained in the qualitative synthesis and risk-of-bias assessment.

Most trials were designed as double-blind randomized controlled studies and were conducted across diverse geographical regions, including Asia, Europe, Africa, and the Americas. The included surgical procedures were heterogeneous and encompassed spine, laparoscopic, ear-nose-throat (ENT), urologic, abdominal, vascular, gynecologic, and breast surgeries.

Sample sizes varied substantially, ranging from 36 to nearly 300 participants per study. Participant characteristics were generally comparable between magnesium and control groups. According to Supplementary Table S2, the mean age of participants in the magnesium groups ranged from 29.31 to 74.93 years, with similar distributions observed in the control groups. Most study populations consisted of middle-aged adults; however, some trials enrolled younger patients undergoing minor or cosmetic procedures (e.g., rhinoplasty), whereas others included predominantly elderly populations, particularly in vascular and urologic surgery.

Sex distribution varied according to surgical type. Several studies included exclusively female populations (e.g., gynecologic surgery, mastectomy), while others enrolled exclusively male participants (e.g., robotic radical prostatectomy). The majority of trials included mixed cohorts with generally balanced sex proportions between treatment groups.

Intravenous magnesium was administered using variable dosing regimens, most commonly as a loading dose of 20–50 mg·kg^−1^ followed by an intraoperative infusion of 2–25 mg·kg^−1^·h^−1^. In some studies, bolus-only or infusion-only protocols were employed. Postoperative analgesia was typically multimodal, most frequently involving opioid-based patient-controlled analgesia (PCA) in combination with non-opioid adjuncts such as paracetamol, non-steroidal anti-inflammatory drugs, nefopam, or tramadol.

Trial registration was reported for a subset of studies, including entries in ClinicalTrials.gov, the Iranian Registry of Clinical Trials (IRCT), the Clinical Research Information Service (CRIS), the Pan African Clinical Trials Registry (PACTR), the Chinese Clinical Trial Registry (ChiCTR), and the Japan Registry of Clinical Trials (jRCT), whereas the remaining trials did not provide registration details.

### 3.4. Primary Outcomes

Early postoperative pain (0–6 h) was reported in 12 randomized controlled trials included in the primary analysis. Intravenous magnesium was associated with a statistically significant reduction in early postoperative pain compared with control (mean difference [MD] −0.90, 95% CI −1.33 to −0.47; *p* < 0.001). However, considerable between-study heterogeneity was observed (I^2^ = 96.27%; τ^2^ = 0.40; Cochran’s Q *p* < 0.001). The 95% prediction interval ranged from −2.39 to 0.59, indicating that future studies may observe smaller or even null effects.

Pain at 12 h was reported in five randomized controlled trials included in the primary analysis. No statistically significant difference was observed between intravenous magnesium and control (mean difference [MD] −0.52, 95% CI −1.15 to 0.11; *p* = 0.10). Substantial between-study heterogeneity was present (I^2^ = 77.38%; τ^2^ = 0.38; Cochran’s Q *p* = 0.001). The 95% prediction interval ranged from −2.73 to 1.69, indicating substantial uncertainty and implying that future studies could yield effects ranging from benefit to no effect or possible harm.

Postoperative pain at 24 h was reported in eight randomized controlled trials included in the primary analysis. Intravenous magnesium was associated with a statistically significant reduction in pain intensity compared with control (mean difference [MD] −0.59, 95% CI −1.00 to −0.18; *p* = 0.0048). Moderate heterogeneity was observed (I^2^ = 66.36%; τ^2^ = 0.20; Cochran’s Q *p* = 0.004). The 95% prediction interval ranged from −1.80 to 0.62, suggesting that although the pooled effect favoured magnesium, future studies may observe smaller or null effects.

**Figure 3:**
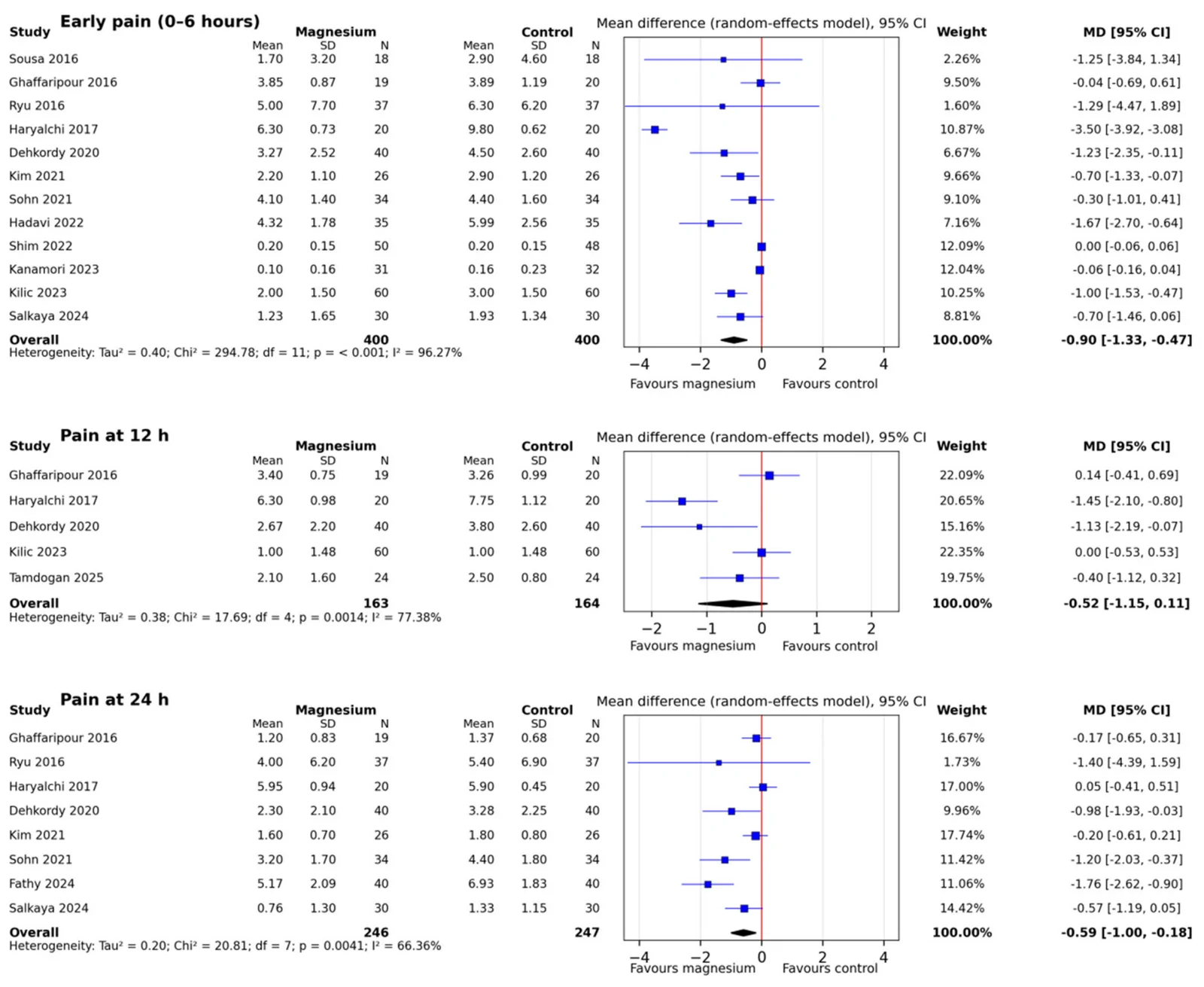
Postoperative pain at different time points (primary analyses). Forest plot comparing intravenous magnesium with control for early postoperative pain (0–6 h) [26,33,34,35,37,40,41,42,44,46,47,51], pain at 12 h [26,35,37,47,55], and pain at 24 h [26,34,35,37,40,41,50,51]. Data are presented as mean differences (MDs) with 95% confidence intervals using a random-effects model. Negative values favour magnesium. Square sizes are proportional to study weight, and diamonds represent pooled effect estimates. Heterogeneity statistics are provided for each outcome.

Pooled estimates derived from random-effects meta-analyses for postoperative pain at different time points are shown in Figure 3.

### 3.5. Secondary Outcomes

#### 3.5.1. Postoperative Opioid Consumption

**Figure 4:**
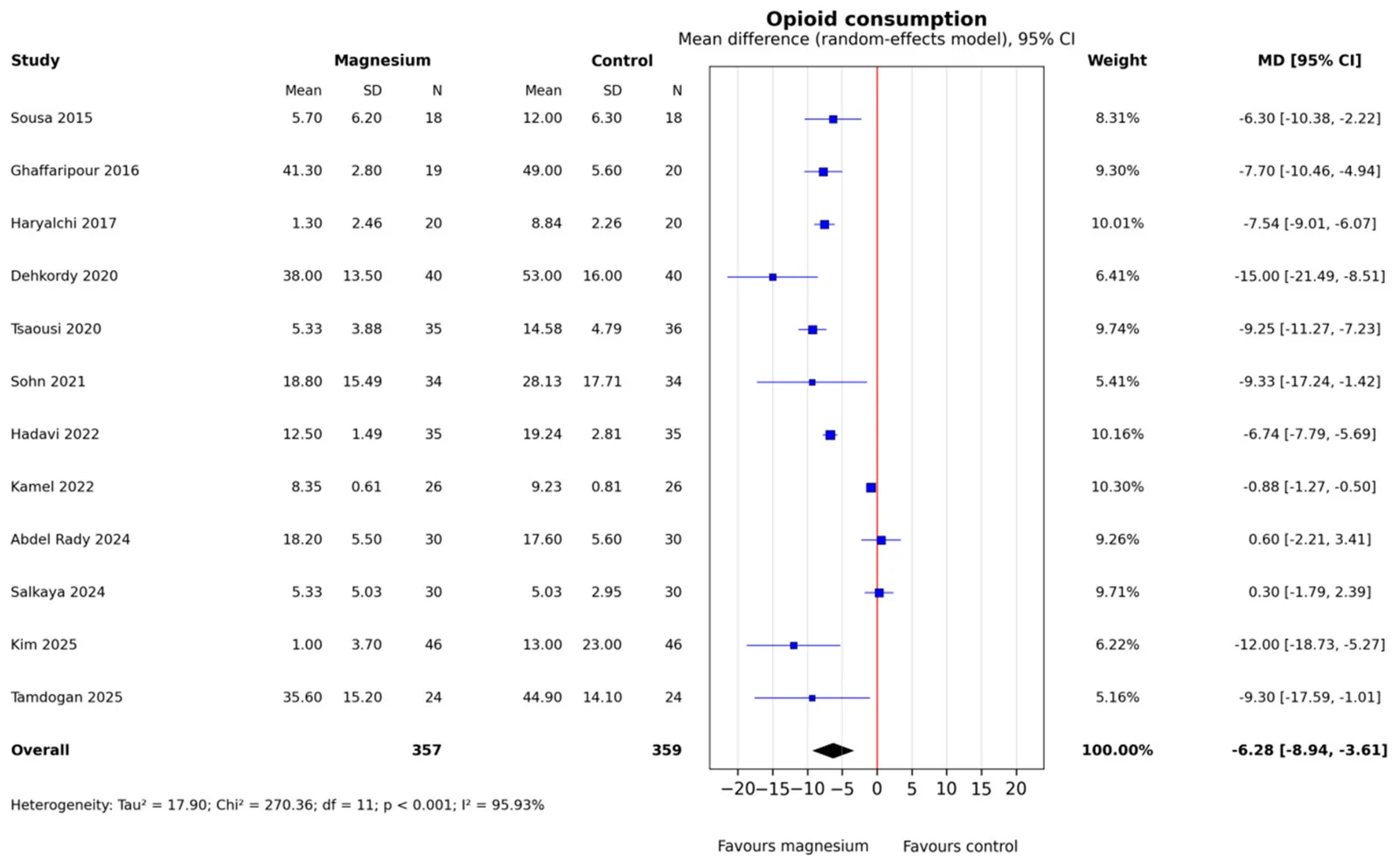
Opioid consumption (IME). Forest plot of postoperative opioid consumption comparing intravenous magnesium with control. Data are presented as mean difference (MD) with 95% confidence intervals (CI) using a random-effects model and expressed as intravenous morphine equivalents (IME). Negative values favour magnesium. Square sizes are proportional to study weight; horizontal lines represent 95% CIs; the diamond indicates the pooled effect estimate. Considerable between-study heterogeneity was observed (I^2^ = 95.93%). Mean ± SD and sample sizes are presented for each group [26,33,35,37,39,41,42,43,49,51,53,55].

Postoperative opioid consumption was reported in 12 randomized controlled trials included in the primary analysis (Figure 4). Intravenous magnesium was associated with a statistically significant reduction in opioid requirements compared with control (mean difference [MD] −6.28 mg morphine equivalents, 95% CI −8.94 to −3.61; *p* < 0.001). Considerable between-study heterogeneity was observed (I^2^ = 95.93%; τ^2^ = 17.90; Cochran’s Q *p* < 0.001).

The 95% prediction interval ranged from −16.18 to 3.62 mg morphine equivalents, indicating that although the pooled estimate favoured magnesium, future studies may observe smaller or null opioid-sparing effects. Despite this heterogeneity, the direction of effect consistently favoured magnesium across the majority of included studies, indicating a robust opioid-sparing effect.

All raw postoperative opioid consumption data, including original units and reporting formats, are provided in Supplementary Table S3.

#### 3.5.2. Time to First Rescue Analgesia

**Figure 5:**
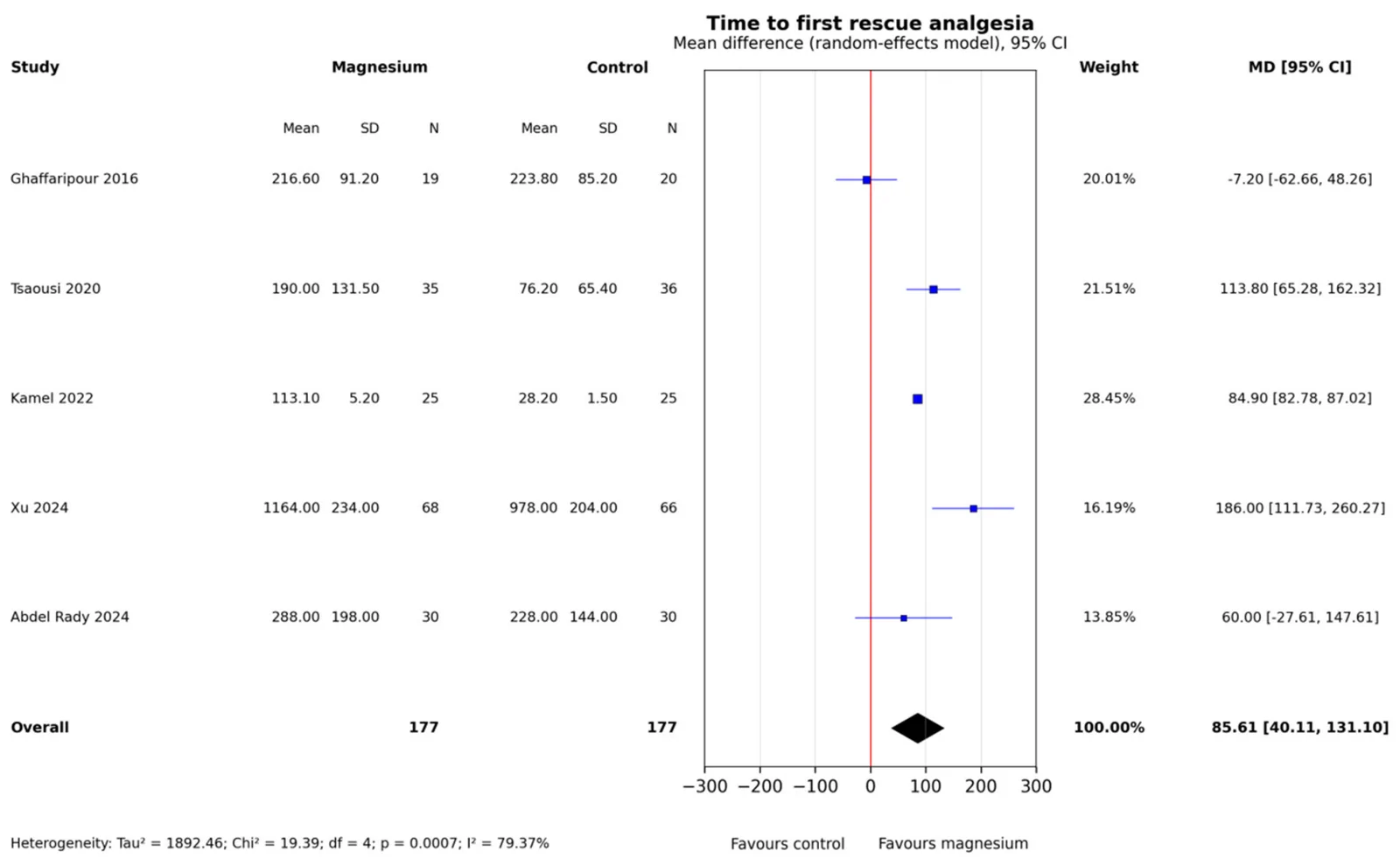
Time to first rescue analgesia. Forest plot of time to first rescue analgesia comparing intravenous magnesium with control. Effect estimates are presented as mean differences (MDs) with 95% confidence intervals (CIs) using a random-effects model. Positive values favour magnesium (longer time to analgesic request). Square sizes are proportional to study weights; horizontal lines represent 95% CIs; the diamond indicates the pooled effect estimate. Substantial between-study heterogeneity was observed (I^2^ = 79.37%). Mean ± SD and sample sizes are provided for each group [26,39,43,49,52].

Time to first rescue analgesia was reported in five randomized controlled trials included in the primary analysis (Figure 5). Intravenous magnesium was associated with a statistically significant prolongation of time to first analgesic request compared with control (mean difference [MD] 85.61 min, 95% CI 40.11 to 131.10; *p* < 0.001). Substantial between-study heterogeneity was observed (I^2^ = 79.37%; τ^2^ = 1892.46; Cochran’s Q *p* < 0.001). The 95% prediction interval ranged from −71.31 to 242.53 min, indicating marked between-study variability and suggesting that future trials may find little or no delay, or alternatively a marked prolongation, in time to rescue analgesia. Nevertheless, the direction of effect was consistent across most studies, indicating a possible clinical benefit of intravenous magnesium in prolonging postoperative analgesia.

#### 3.5.3. Sensitivity Analyses

Sensitivity analyses were conducted to evaluate the robustness of the primary findings by incorporating studies at high risk of bias and those with data extracted from graphical presentations (Supplementary Figures S2–S4). For early postoperative pain (0–6 h), inclusion of these studies attenuated the treatment effect, but it remained statistically significant (MD −0.51, 95% CI −0.80 to −0.22; *p* < 0.001; I^2^ = 95.33%). For pain at 12 h, the sensitivity analysis demonstrated a statistically significant reduction in pain (MD −0.82, 95% CI −1.35 to −0.29; *p* = 0.002; I^2^ = 92.69%), whereas the primary analysis was not significant. For pain at 24 h, the findings were consistent with the primary analysis (MD −0.54, 95% CI −0.77 to −0.31; *p* < 0.001; I^2^ = 65.40%). Overall, these analyses supported the robustness of the analgesic effect of intravenous magnesium, particularly for early postoperative pain and pain at 24 h.

#### 3.5.4. Publication Bias Analyses

**Figure 6:**
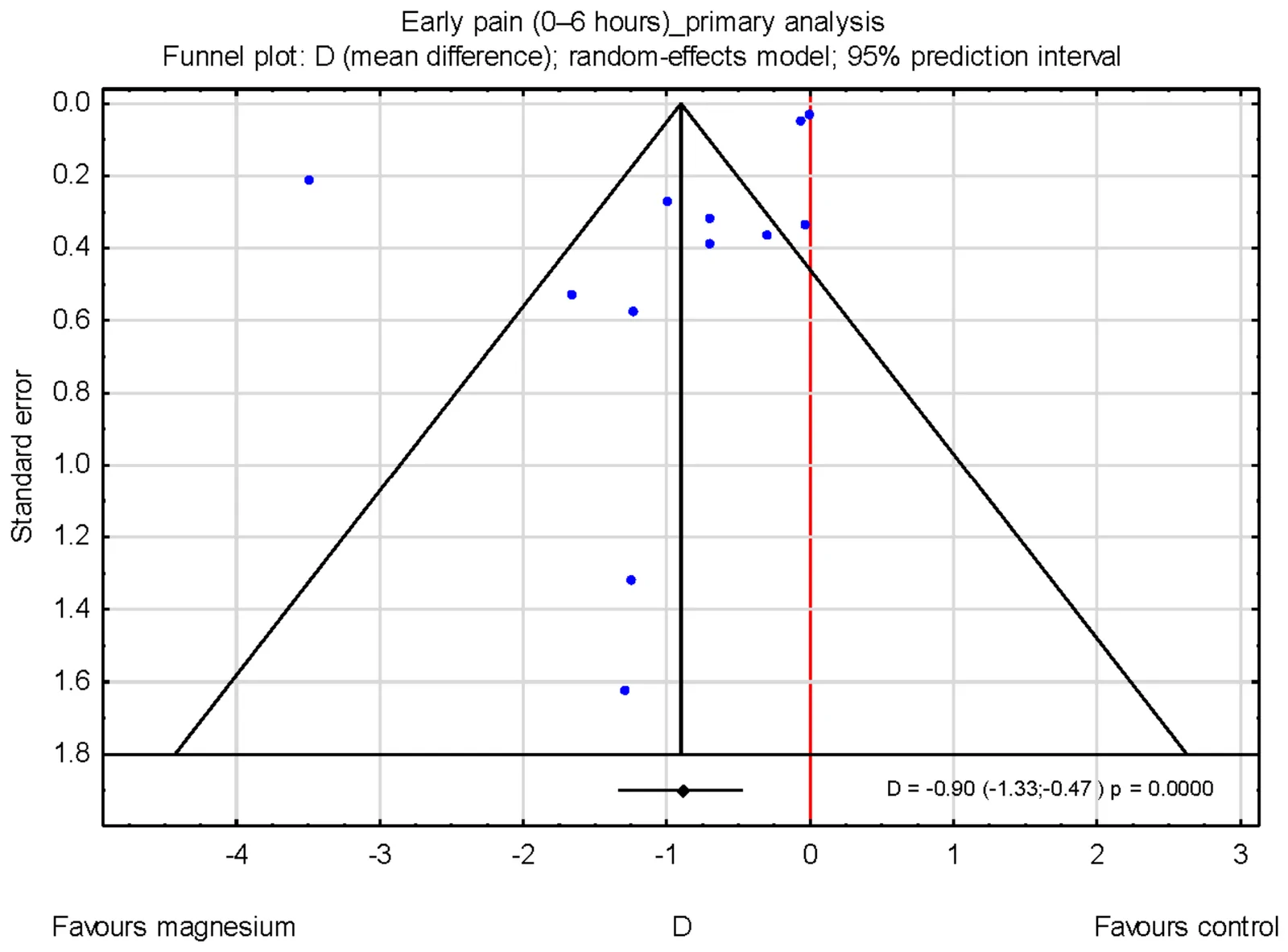
Funnel plot for early postoperative pain (0–6 h). Mean differences (MDs) are plotted against standard error. The vertical line represents the pooled effect estimate, and the triangular region represents the expected 95% confidence limits. Negative values favour magnesium. Formal tests for funnel plot asymmetry were not statistically significant: Egger’s test *p* = 0.07; Begg’s test *p* = 0.53.

Assessment of publication bias was restricted to outcomes including at least 10 studies. Funnel plots for early postoperative pain and opioid consumption are presented in Figure 6 and Supplementary Figure S5, respectively.

For early postoperative pain, formal tests for funnel plot asymmetry were not statistically significant (Egger’s test *p* = 0.07; Begg’s test *p* = 0.53), suggesting no statistically significant evidence of small-study effects, although the possibility cannot be excluded. In contrast, for opioid consumption, visual inspection suggested asymmetry, and Egger’s test indicated possible small-study effects (*p* = 0.03), whereas Begg’s test was not statistically significant (*p* = 0.65).

### 3.6. Grade Assessment

The certainty of evidence varied across outcomes (Table 2). For primary outcomes, certainty ranged from very low to moderate, with moderate certainty for pain at 24 h, low certainty for early postoperative pain, and very low certainty for pain at 12 h. For secondary outcomes, certainty was very low for opioid consumption and low for time to first rescue analgesia.

### 3.7. Meta-Regression Analyses

Subgroup effects were formally tested using random-effects meta-regression with binary moderators. No significant effect modification was observed for either early postoperative pain or pain at 24 h across timing of magnesium administration (early pain: b = −0.02; *p* = 0.98; 24 h: b = 0.16; *p* = 0.77), loading dose (>30 vs. ≤30 mg·kg^−1^; early pain: b = 0.35; *p* = 0.70; 24 h: b = 0.07; *p* = 0.90), surgical type (early pain: b = 0.59; *p* = 0.213; 24 h: b = −0.70; *p* = 0.07), or trial registration status (early pain: b = 0.11; *p* = 0.82; 24 h: b = 0.19; *p* = 0.71).

Publication period (≤2020 vs. >2020) significantly modified the treatment effect for early postoperative pain (b = 1.33, SE = 0.35, 95% CI 0.65 to 2.00; *p* < 0.001; R^2^ = 62.1%), whereas no significant effect modification was observed at 24 h (b = −0.51; *p* = 0.23).

Exploratory meta-regression using continuous moderators showed no significant association between mean patient age and treatment effect for either early postoperative pain (b = 0.04, SE = 0.02, 95% CI −0.00 to 0.08; *p* = 0.07; R^2^ = 0.0%) or pain at 24 h (b = 0.02, SE = 0.03, 95% CI −0.05 to 0.08; *p* = 0.61; R^2^ = 0.0%). Similarly, magnesium loading dose was not associated with treatment effect for early postoperative pain (b = 0.00, SE = 0.02, 95% CI −0.04 to 0.04; *p* = 0.90; R^2^ = 0.0%) or pain at 24 h (b = 0.02, SE = 0.03, 95% CI −0.05 to 0.08; *p* = 0.59; R^2^ = 0.0%).

The proportion of female participants significantly modified the treatment effect on early postoperative pain (b = −2.58, SE = 0.51, 95% CI −3.58 to −1.59; *p* < 0.001; R^2^ = 59.6%), with greater analgesic effects observed in studies including a higher proportion of female participants. No significant effect modification was observed at 24 h (b = 0.49; *p* = 0.54).

Because of the limited number of contributing studies, the meta-regression findings warrant cautious interpretation. Detailed study-level moderator data are presented in Supplementary Table S4, and full meta-regression outputs are presented in Supplementary Table S5.

## 4. Discussion

This review updates the evidence on perioperative intravenous magnesium by focusing on trials published from 2015 onward, a period when multimodal postoperative analgesia and ERAS protocols had become increasingly common [1,6]. The pooled estimates favored magnesium for early postoperative pain and pain at 24 h, and also showed lower opioid consumption and a longer time to first rescue analgesia. However, the results were not uniform. The 12 h estimate was statistically uncertain, and early pain and opioid-related outcomes showed considerable heterogeneity. These findings point to magnesium more as an opioid-sparing adjunct within multimodal analgesia than as a stand-alone intervention with a predictable effect on pain intensity.

On a 0–10 pain scale, the pooled reductions for early pain (MD −0.90) and pain at 24 h (MD −0.59) remain below the conventional threshold for an individually perceptible analgesic benefit [56]. Therefore, the clinical relevance lies in the accompanying reduction in opioid requirements and the prolongation of time to rescue analgesia. In practice, magnesium may therefore be useful even when its direct effect on pain scores is weak, provided the aim is to reduce opioid exposure as part of a broader analgesic strategy.

Our findings are consistent with previous meta-analyses reporting analgesic and opioid-sparing effects of systemic magnesium [11,12]. The present synthesis, limited to contemporary randomized trials, suggests a subtler effect. Modern perioperative care already includes non-opioid analgesics, protocolized rescue analgesia, and regional or local techniques [6,18]. Given the myriad clinical and surgical scenarios, the incremental benefit of magnesium is likely to be more dependent on the surgical model and background analgesic regimen than earlier pooled estimates might suggest.

The meta-regression findings should be read as exploratory rather than definitive. Timing of administration, loading dose, surgical category, and trial registration status did not explain the observed between-study variation, and there was no clear dose–response relationship. The associations between early pain effects and publication period or the proportion of female participants may reflect differences in surgical cohorts, baseline analgesic protocols, or chance findings from a limited number of study-level observations.

Prediction intervals are particularly important in this review [23]. For several outcomes, they crossed or approached no effect, meaning that a future trial in a different perioperative setting could show little or no benefit despite a favorable pooled mean, thereby limiting the strength of a general recommendation. Magnesium appears most useful when applied selectively in patients or procedures where opioid reduction is a priority, rather than as a universal adjunct for all adults undergoing surgery under general anesthesia.

The GRADE assessment supports this restrained interpretation. Certainty was highest for pain at 24 h and lower for early pain, opioid consumption, and time to first rescue analgesia, mainly because of inconsistency and imprecision. Overall, the evidence supports a potential role for intravenous magnesium in multimodal postoperative analgesia, but the expected benefit is context-dependent and probably modest.

## 5. Strengths and Limitations

A strength of this review is based on contemporary randomized controlled trials, making the findings more applicable to current practice than syntheses dominated by older studies. The review also used a registered protocol, PRISMA-based reporting, outcome-level RoB 2 assessment, and GRADE evaluation. Standardizing pain scores to a common 0–10 metric and converting opioid doses to intravenous morphine equivalents improved comparability across trials, while sensitivity analyses and meta-regression helped test the stability of the main findings.

The main limitation is the clinical and methodological diversity of the included trials. Studies differed in surgical procedure, magnesium dose and timing, background analgesia, outcome definitions, and reporting format. As a result, the pooled estimates should be interpreted as average effects across heterogeneous perioperative contexts, not as a fixed treatment effect expected in every clinical setting [57]. Some studies required transforming medians and interquartile ranges or extracting values from figures, which introduces uncertainty despite the use of established methods [27,28]. In one study [26], opioid consumption was reported in mg/kg rather than as an absolute dose and was converted to milligrams assuming a standard adult body weight of 70 kg. Although this assumption may have introduced a small degree of imprecision, it affected only a single study and is therefore unlikely to have materially influenced the pooled estimate. Egger’s test suggested possible small-study effects for postoperative opioid consumption. Therefore, publication bias or other small-study effects cannot be excluded, and the observed opioid-sparing effect should be interpreted with caution [32]. In addition, although meta-regression analyses were prespecified, they were based on a limited number of studies and should therefore be considered exploratory and interpreted with caution. Publication bias was also suspected for opioid consumption [32]. Finally, this review did not provide a detailed synthesis of adverse events or serum magnesium concentrations, so the balance between analgesic benefit and safety could not be fully evaluated.

## 6. Conclusions

Among contemporary randomized controlled trials, intravenous magnesium sulfate was associated with little reduction in postoperative pain and with opioid-sparing outcomes. The pain-score reductions were small, and the substantial heterogeneity means that the pooled estimates should not be translated into a single expected benefit for individual patients. The results support considering magnesium as an adjunct within multimodal analgesia, particularly when reducing postoperative opioid use is clinically relevant. Further trials should identify which surgical populations, dosing schedules, and background analgesic regimens produce a clinically meaningful benefit.

## Figures and Tables

**Table 1:** Characteristics of included studies [26,33,34,35,36,37,38,39,40,41,42,43,44,45,46,47,48,49,50,51,52,53,54,55].

Study (Year)	Country	Surgery	Design	N (Mg/C)	Magnesium Regimen	Postoperative Analgesia	Pain Scale	Variables
Sousa AM et al., 2016 [33]	Brazil	Laparoscopic gynecologic	DB-RCT	18/18	20 mg·kg^−1^ + 2 mg·kg^−1^·h^−1^	PCA morphine + IV dipyrone	VAS (0–100)	①† ④
Ghaffaripour S. et al., 2016 [26]	Iran	Laminectomy	DB-RCT	19/20	30 mg·kg^−1^ + 10 mg·kg^−1^·h^−1^	Morphine protocol	VAS (0–100)	① ② ③ ④ ⑤
Ryu J.H et al., 2016 [34]	South Korea	Laparoscopic gastrectomy	DB-RCT	37/37	50 mg·kg^−1^ + 15 mg·kg^−1^·h^−1^	PCA fentanyl	NRS (0–100)	①† ③†
Haryalchi K. et al., 2017 [35]	Iran	Total abdominal hysterectomy	DB-RCT	20/20	15 mg·kg^−1^·h^−1^	Pethidine	VNRS (0–10)	① ② ③ ④
Elsersy H.E et al., 2017 [36]	Egypt	Endoscopic sinus	DB-RCT	146/148	30 mg·kg^−1^ + 9 mg·kg^−1^·h^−1^	IV paracetamol + IV pethidine PRN	NRS (0–10)	④
Dehkordy ME. et al., 2020 [37]	Iran	Posterior lumbar spinal fusion surgery	DB-RCT	40/40	50 mg/kg^−1^ + 15 mg·kg^−1^·h^−1^	PCA morphine with ondansetron + meperidine PRN	VAS (0–100)	① ② ③ ④
Moon S. et al., 2020 [38]	South Korea	Laparoscopic gynecologic	DB-RCT	31/30	40 mg·kg^−1^ + 10 mg·kg^−1^·h^−1^	PCA fentanyl + ketorolac PRN	NRS (0–10)	③*† ④*
Tsaousi G. et al., 2020 [39]	Greece	Lumbar laminectomy	DB-RCT	35/36	20 mg·kg^−1^ + 20 mg·kg^−1^·h^−1^	IV paracetamol + lornoxicam + morphine PRN	VAS (0–10)	④ ⑤
Kim H.Y. et al., 2021 [40]	South Korea	Robotic radical prostatectomy	DB-RCT	26/26	50 mg kg^−1^ + 10 mg kg^−1^·h^−1^	PCA fentanyl + IV nefopam + IV ibuprofen + acetaminophen orally	VAS (0–10)	① ③
Sohn M.H. et al., 2021 [41]	South Korea	Spine surgery	DB-RCT	34/34	30 mg·kg^−1^ + 15 mg·kg^−1^·h^−1^	PCA fentanyl + morphine/propacetamol/meperidine/fentanyl/acetaminophen/ketorolac/nefopam/ketorolac PRN	NRS (0–10)	① ③ ④
Hadavi S.M.R. et al., 2022 [42]	Iran	Rhinoplasty	DB-RCT	35/35	30 mg·kg^−1^	IV morphine protocol	NRS (0–10)	① ④
Kamel A.A.F. et al., 2022 [43]	Egypt	Nasal surgery	DB-RCT	25/25	40 mg·kg^−1^ + 10–15 mg·kg^−1^·h^−1^	IV paracetamol + IV pethidine PRN	VAS (0–10)	①*④ ⑤
Shim J.W. et al., 2022 [44]	South Korea	Transurethral resection of bladder tumor	DB-RCT	48/50	50 mg·kg^−1^ + 15 mg·kg^−1^·h^−1^	PACU fentanyl + tramadol PRN	VAS (0–100)	①†
Jiang W. et al., 2023 [45]	China	Laparoscopic radical resection of gastrointestinal cancer	DB-RCT	45/43	40 mg·kg^−1^ + 15 mg·kg^−1^·h^−1^	IV Sufentanil + IV morphine PRN	NRS (0–10)	‡ clearly skewed data
Kanamori H. et al., 2023 [46]	Japan	Endovascular repair of aortic aneurysm	DB-RCT	31/32	30 mg·kg^−1^ + 10 mg·kg^−1^·h^−1^	IV paracetamol	NRS (0–10)	①†
Kilic K. et al., 2023 [47]	Turkey	Septorhinoplasty	RCT	60/60	30 mg·kg^−1^ + 9 mg·kg^−1^·h^−1^	IV dexketoprofen + tramadol PRN	VAS (0–10)	①† ②†
Su Y.H. et al., 2023 [48]	China	Radical mastectomy	DB-RCT	35/35	30 mg·kg^−1^ + 10 mg·kg^−1^·h^−1^	IV tramadol PRN	VAS (0–10)	‡ clearly skewed data
Abdel Rady M.M. et al., 2024 [49]	Egypt	Spine fusion	DB-RCT	30/30	30 mg·kg^−1^	IV paracetamol + IV ketorolac + IV morphine PRN	NRS (0–10)	①*②*③* ④ ⑤
Fathy W. et al., 2024 [50]	Egypt	Lumbar fixation	RCT	40/40	30 mg·kg^−1^ + 10 mg·kg^−1^·h^−1^	IM diclofenac + IV paracetamol	VAS (0–10)	③
Salkaya A. et al., 2024 [51]	Turkey	Lumbar surgery	DB-RCT	30/30	10 mg·kg^−1^·h^−1^	PCA pethidine + diclofenac PRN	VAS (0–10)	① ③ ④
Xu H. et al., 2024 [52]	China	Total knee arthroplasty	DB-RCT	68/66	40 mg·kg^−1^ + 15 mg·kg^−1^·h^−1^	PCA fentanyl + Femoral nerve block	NRS (0–10)	⑤
Kim R.K. et al. 2025 [53]	USA	Peroral endoscopic esophageal myotomy	DB-RCT	46/46	50 mg·kg^−1^ + 25 mg·kg^−1^·h^−1^	Fentanyl/ hydromorphone PRN	NRS (0–10)	④
Nashibi M. et al., 2025 [54]	Iran	Spinal fusion	DB-RCT	20/20	50 mg·kg^−1^ + 15 mg·kg^−1^·h^−1^	IV ketorolac PRN + IV pethidine PRN	NRS (0–10)	① ② ③
Tamdogan I. et al., 2025 [55]	Turkey	Abdominal hysterectomy	DB-RCT	24/24	20 mg·kg^−1^ + 20 mg·kg^−1^·h^−1^	IV paracetamol + IV ketoprofen + PCA fentanyl + IV tramadol PRN	NRS (0–10)	③† ④

Footnotes: ① Early pain defined as one representative timepoint within 0–6 h postoperatively (hierarchy: 6 h → 4 h → 2 h → 1 h → PACU/0 h). ② Pain at 12 ③ Pain at 24 ④ 24 h opioid consumption ⑤ Time to rescue analgesia * Data extracted from graphical presentation. † Mean and SD reconstructed from non-parametric data. ‡ Study included in the qualitative synthesis but not in the quantitative meta-analysis due to non-convertible data. Abbreviations: DB-RCT—double-blind randomized controlled trial; TB-RCT—triple-blind randomized controlled trial; RCT—randomized controlled trial; VAS—Visual Analogue Scale; NRS—Numeric Rating Scale; NPRS—Numeric Pain Rating Scale; VNRS—Verbal Numeric Rating Scale; PCA—patient-controlled analgesia; PRN—pro re nata (as needed); IV—intravenous; IM—intramuscular; PACU—post-anesthesia care unit.

**Table 2:** GRADE assessment of the certainty of evidence for primary and secondary outcomes.

Outcome	No. of Studies (Participants)	Effect (95% CI)	Certainty	Rating	Reasons for Downgrading
Primary outcomes					
Early postoperative pain (0–6 h)	12 RCTs (800)	MD −0.90 (−1.33 to −0.47)	Low	⨁⨁◯◯	Serious inconsistency (I^2^ = 96.27%); possible publication bias cannot be excluded
Pain at 12 h	5 RCTs (467)	MD −0.52 (−1.15 to 0.11)	Very low	⨁◯◯◯	Serious inconsistency (I^2^ = 77.38%); very serious imprecision (confidence interval crosses no effect)
Pain at 24 h	8 RCTs (493)	MD −0.59 (−1.00 to −0.18)	Moderate	⨁⨁⨁◯	Serious inconsistency (I^2^ = 66.36%)
Secondary outcomes					
Opioid consumption	12 RCTs (716)	MD −6.28 (−8.94 to −3.61)	Very low	⨁◯◯◯	Very serious inconsistency (I^2^ = 95.93%); suspected publication bias (Egger *p* = 0.03)
Time to first rescue analgesia	5 RCTs (354)	MD +85.61 (40.11 to 131.10)	Low	⨁⨁◯◯	Serious inconsistency (I^2^ = 79.37%); serious imprecision

Abbreviations: CI, confidence interval; GRADE, Grading of Recommendations Assessment, Development and Evaluation; MD, mean difference; RCT, randomized controlled trial. Evidence from randomized controlled trials starts at high certainty and was downgraded based on risk of bias, inconsistency, indirectness, imprecision, and publication bias. Risk of bias was not downgraded overall, as most studies were rated as low risk of bias or some concerns. Heterogeneity was substantial or considerable across most outcomes. Publication bias assessment was feasible for early pain and opioid consumption only.

## Data Availability

No new data were created or analyzed in this study. Data sharing is not applicable to this article.

## Supplementary Materials

The following supporting information can be downloaded at https://www.mdpi.com/article/10.3390/ph19081296/s1; Figure S1: Percentage distribution of risk-of-bias judgments across RoB 2 domains; Figure S2: Sensitivity analysis—early pain; Figure S3: Sensitivity analysis—pain at 12 h; Figure S4: Sensitivity analysis—pain at 24 h; Figure S5: Funnel plot for postoperative opioid consumption at 24 h; Table S1: PRISMA_2020_checklist; Table S2: Study characteristics and participant demographics, including trial registration; Table S3: Raw postoperative opioid consumption data across included studies; Table S4: Study-level moderators used in meta-regression analyses; Table S5: Results of exploratory random-effects meta-regression analyses.
