# Supplementary material for: Effect of Intravenous Magnesium on Postoperative Pain and Analgesic Outcomes in Patients Undergoing General Anesthesia: A Systematic Review and Meta-Analysis of Randomized Controlled Trials
Source: Pharmaceuticals (Basel). 2026 Aug 16;19(8):1296. doi: 10.3390/ph19081296 (PMC13516737; doi:10.3390/ph19081296)
Supplement: Supplementary file 1 [file pharmaceuticals-19-01296-s001.zip › pharmaceuticals-4465062-supplementary.pdf]

Supplementary figures

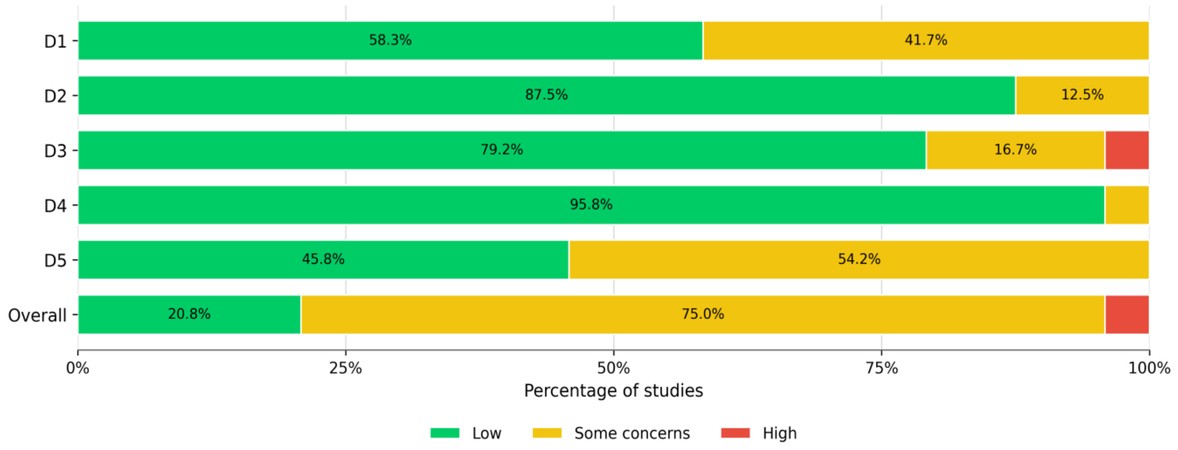

Supplementary Figure S1. Percentage distribution of risk-of-bias judgments across RoB 2 domains.

Stacked bar chart showing the percentage distribution of risk-of-bias judgments across the five RoB 2 domains (D1–D5) and the overall risk-of-bias assessment for all included randomized controlled trials. Green indicates low risk of bias, yellow indicates some concerns, and red indicates high risk of bias.

Intravenous magnesium sulfate was associated with a reduction in postoperative pain, particularly at 24 h, as well as with significant opioid-sparing effects and prolongation of time to first rescue analgesia. Although the magnitude of pain reduction was modest, the overall findings support the incorporation of magnesium into multimodal analgesic strategies for appropriately selected patients undergoing surgery under general anesthesia. However, the presence of substantial heterogeneity and the generally low-to-moderate certainty of evidence warrant cautious interpretation. Future high-quality randomized controlled trials are needed to define the optimal dosing, timing, and patient populations most likely to benefit from intravenous magnesium.

Early pain (0–6 hours) (sensitivity analysis)

Mean difference (random-effects model), 95% CI

Study	Magnesium Mean	Magnesium SD	Magnesium N	Control Mean	Control SD	Control N	Weight	MD [95% CI]
Sousa 2016	1.70	3.20	18	2.90	4.60	18	1.11%	-1.25 [-3.84, 1.34]
Ghaffaripour 2016	3.85	0.87	19	3.89	1.19	20	6.40%	-0.04 [-0.69, 0.61]
Ryu 2016	5.00	7.70	37	6.30	6.17	37	0.77%	-1.29 [-4.47, 1.89]
Haryalchi 2017	6.30	0.73	20	9.80	0.62	20	7.89%	-3.50 [-3.92, -3.08]
Dehkordy 2020	3.27	2.52	40	4.50	2.60	40	3.93%	-1.23 [-2.35, -0.11]
Kim 2021	2.20	1.10	26	2.90	1.20	26	6.57%	-0.70 [-1.33, -0.07]
Sohn 2021	4.10	1.40	34	4.40	1.60	34	6.01%	-0.30 [-1.01, 0.41]
Hadavi 2022	4.32	1.78	35	5.99	2.56	35	4.31%	-1.67 [-2.70, -0.64]
Kamel 2022*	2.00	0.60	25	1.90	0.50	25	8.54%	0.10 [-0.21, 0.41]
Shim 2022	0.20	0.15	50	0.20	0.15	48	9.40%	0.00 [-0.06, 0.06]
Kanamori 2023	0.10	0.16	31	0.16	0.23	32	9.34%	-0.06 [-0.16, 0.04]
Kilic 2023	2.00	1.48	60	3.00	1.48	60	7.19%	-1.00 [-1.53, -0.47]
Abdel Rady 2024*	2.70	0.20	30	2.90	0.20	30	9.34%	-0.20 [-0.30, -0.10]
Salkaya 2024	1.23	1.65	30	1.93	1.34	30	5.74%	-0.70 [-1.46, 0.06]
Nashibi (2025a)**	5.45	0.92	20	4.45	0.76	20	7.24%	1.00 [0.48, 1.52]
Nashibi (2025b)**	5.00	1.36	20	4.45	0.76	20	6.22%	0.55 [-0.13, 1.23]
**Overall**			**495**			**495**	**100.00%**	**-0.51 [-0.80, -0.22]**

Heterogeneity: Tau² = 0.23; Chi² = 321.41; df = 15; p = < 0.001; I² = 95.33%

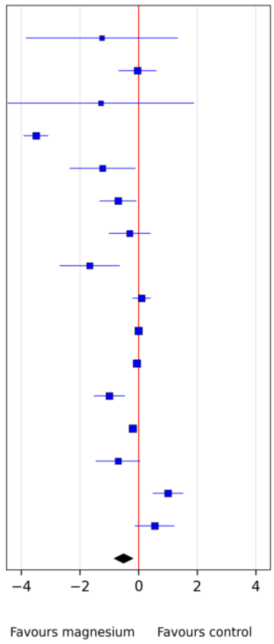

Supplementary Figure S2. Sensitivity analysis – early pain

Forest plot of the sensitivity analysis for early postoperative pain intensity. The analysis included all eligible studies, including those with data estimated from graphical reports (*) or those at high risk of bias (**). Effect estimates are presented as mean differences (MDs) with 95% confidence intervals (CIs) using a random-effects model. Negative values favour magnesium. Square sizes are proportional to study weights; horizontal lines represent 95% CIs; the diamond indicates the pooled effect estimate. Heterogeneity remained considerable (I^2 = 95.33%). Mean ± SD and sample sizes are provided for both groups.

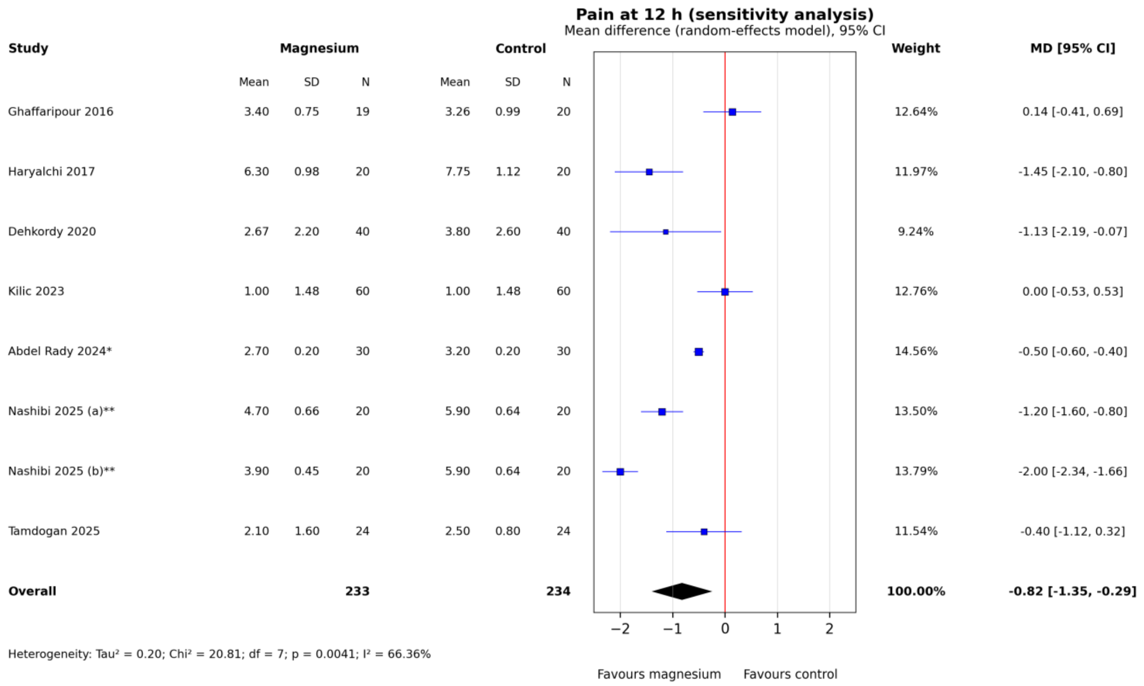

Supplementary Figure S3. Sensitivity analysis – pain at 12 h

Forest plot of the sensitivity analysis for postoperative pain intensity at 12 h. The analysis included all eligible studies, including those with data estimated from graphical reports (*) or those at high risk of bias (**). Effect estimates are presented as mean differences (MDs) with 95% confidence intervals (CIs) using a random-effects model. Negative values favour magnesium. Square sizes are proportional to study weights; horizontal lines represent 95% CIs; the diamond indicates the pooled effect estimate. Heterogeneity remained considerable (I^2 = 92.69%). Mean ± SD and sample sizes are provided for both groups.

Pain at 24 h (sensitivity analysis)
Mean difference (random-effects model), 95% CI

Study	Magnesium			Control			Weight	MD [95% CI]
	Mean	SD	N	Mean	SD	N		
Ghaffaripour 2016	1.20	0.83	19	1.37	0.68	20	9.62%	-0.17 [-0.65, 0.31]
Ryu 2016	4.00	1.90	37	5.10	2.10	37	4.53%	-1.10 [-2.01, -0.19]
Haryalchi 2017	5.95	0.94	20	5.90	0.45	20	9.98%	0.05 [-0.41, 0.51]
Dehkordy 2020	2.30	2.10	40	3.28	2.25	40	4.24%	-0.98 [-1.93, -0.03]
Moon 2020*	3.30	2.30	31	3.40	2.30	30	3.15%	-0.10 [-1.25, 1.05]
Kim 2021	1.60	0.70	26	1.80	0.80	26	10.86%	-0.20 [-0.61, 0.21]
Sohn 2021	3.20	1.70	34	4.40	1.80	34	5.16%	-1.20 [-2.03, -0.37]
Abdel Rady 2024*	2.40	0.20	30	2.80	0.20	30	16.18%	-0.40 [-0.50, -0.30]
Fathy 2024	5.17	2.09	40	6.93	1.83	40	4.92%	-1.76 [-2.62, -0.90]
Salkaya 2024	0.76	1.30	30	1.33	1.15	30	7.44%	-0.57 [-1.19, 0.05]
Nashibi (2025a)	3.60	0.60	20	4.00	0.65	20	11.27%	-0.40 [-0.79, -0.01]
Nashibi (2025b)	3.10	0.31	20	4.00	0.65	20	12.66%	-0.90 [-1.21, -0.59]
**Overall**			**347**			**347**	**100.00%**	**-0.54 [-0.77, -0.31]**

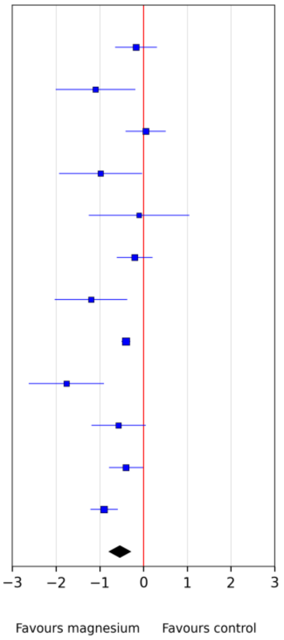

Heterogeneity: Tau² = 0.08; Chi² = 31.79; df = 11; p = 0.0008; I² = 65.40%

Supplementary Figure S4. Sensitivity analysis – pain at 24 h

Forest plot of the sensitivity analysis for postoperative pain intensity at 24 h. The analysis included all eligible studies, including those with data estimated from graphical reports (*) or those at high risk of bias (**). Effect estimates are presented as mean differences (MDs) with 95% confidence intervals (CIs) using a random-effects model. Negative values favour magnesium. Square sizes are proportional to study weights; horizontal lines represent 95% CIs; the diamond indicates the pooled effect estimate. Heterogeneity remained moderate (I^2 = 65.40%). Mean ± SD and sample sizes are provided for both groups.

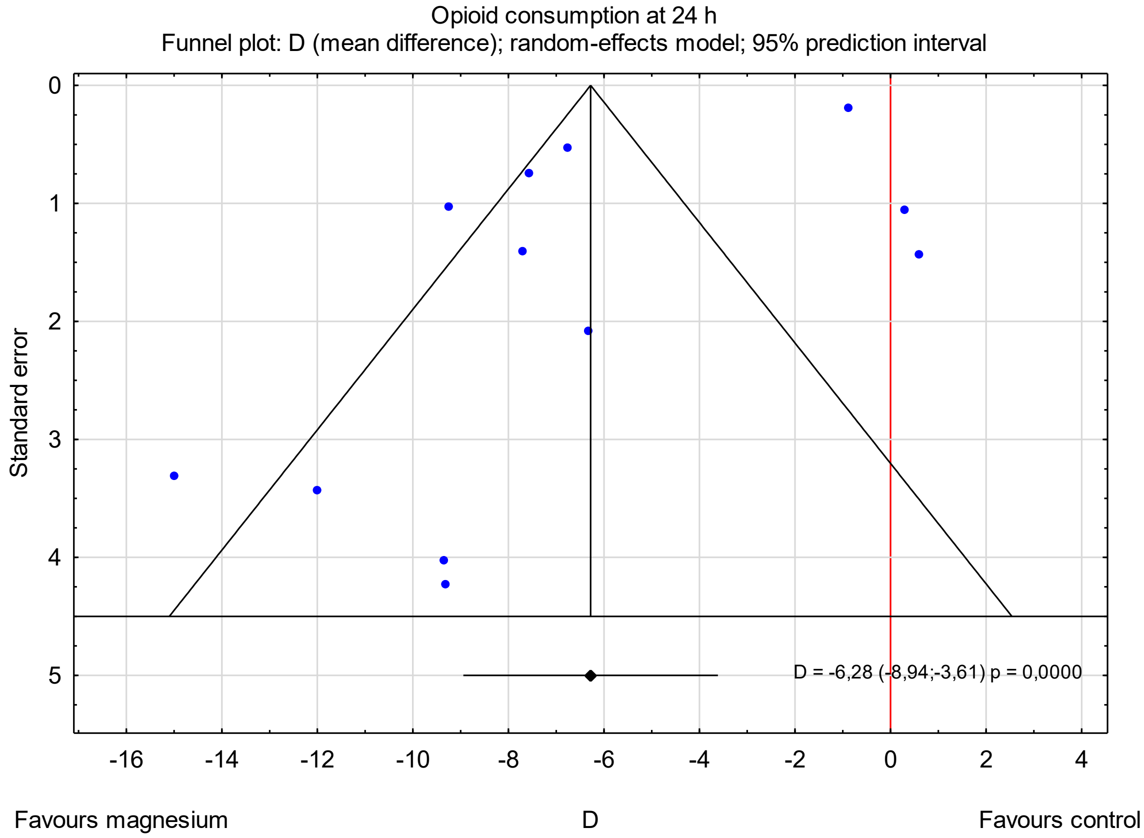

Supplementary Figure S5. Funnel plot for postoperative opioid consumption at 24 h.

Mean differences (MDs) are plotted against standard error. The vertical line represents the pooled effect estimate, and the triangular region represents the expected 95% confidence limits. Negative values favour magnesium. Visual inspection suggests asymmetry. Egger's test indicated potential small-study effects (p = 0.03), whereas Begg's test was not statistically significant (p = 0.65).

Supplementary Table S2. Study characteristics and participant demographics, including trial registration

Study	Registration	N (Mg/C)	Age (Mg)	Age (C)	Sex (Mg)	Sex (C)
Sousa 2016	NR	18/18	49.90 ± 8.10	47.40 ± 13.10	Female	Female
Ghaffaripour 2016	IRCT (2015012019470N12)	19/20	44.7	43.5	8/11	8/12
Ryu 2016	CRIS (KCT0001035)	37/37	52.30 ± 12.00	55.70 ± 9.10	20/17	29/8
Haryalchi 2017	IRCT (2014012316325N1)	20/20	48.30 ± 5.66	50.70 ± 2.20	Female	Female
Elsersy 2017	PACTR (201402000737691)	146/148	36.00 ± 7.00	36.00 ± 8.00	80/66	81/67
Dehkordy 2020	NR	40/40	49.90 ± 12.10	45.70 ± 12.70	17/23	14/26
Tsaousi 2020	ClinicalTrials.gov (NCT04161729)	35/36	55.90 ± 10.80	49.00 ± 15.00	13/22	15/21
Moon 2020	NR	31/30	40.30 ± 11.60	41.90 ± 10.00	Female	Female
Kim 2021	ClinicalTrials.gov (NCT02833038)	26/26	63.00 ± 7.00	65.00 ± 7.00	Male	Male
Sohn 2021	CRIS (KCT0004173)	34/34	56.50 ± 13.70	56.50 ± 14.70	14/20	17/17
Hadavi 2022	IRCT (20121204011662N12)	35/35	29.31 ± 7.94	25.47 ± 4.81	8/27	12/23
Kamel 2022	ClinicalTrials.gov (NCT04688203)	25/25	31.80 ± 5.60	32.90 ± 5.70	14/11	11/12
Shim 2022†	CRIS (KCT0006875)	48/50	66.58 ± 10.70	64.23 ± 14.50	35/13	40/10
Jiang 2023	NR	45/43	61.00 ± 7.10	60.7 ± 8.00	Male	Male
Kanamori 2023†	jRCT (iRCTs041190013)	31/32	74.93 ± 5.44	77.46 ± 7.22	31/1	26/6
Kilic 2023	NR	60/60	NR	NR	20/40	17/43
Su 2023†	ChiCTR2300070595	35/35	52.36 ± 7.00	53.64 ± 8.50	Female	Female
Fathy 2024	ClinicalTrials.gov (NCT05632159)	40/40	46.11 ± 11.89	42.20 ± 10.41	27/13	26/14

Salkaya 2024	NR	30/30	58.40 ± 9.50	60.30 ± 11.00	13/17	12/18
Xu 2024†	ChiCTR2200065940	68/66	67.00 ± 4.54	66.35 ± 6.82	14/54	6/50
Abdel Rady 2024	ClinicalTrials.gov (NCT04776369)	30/30	40.80 ± 11.30	37.20 ± 9.90	18/12	21/9
Tamdogan 2025	NR	24/24	55.90 ± 9.10	52.20 ± 9.10	Female	Female
Kim 2025	NR	46/46	58.00 ± 18.00	59.00 ± 18.00	19/27	19/27
Nashibi 2025	NR	20/20	NR	NR	NR	NR

Abbreviations: Mg, magnesium group; C, control group; NR, not reported. Values are presented as mean (SD) or mean only. Where studies reported medians or ranges, values were converted to mean and SD using established methods (Wan et al., Luo et al.) and are marked with †. Sex is presented as male/female where available.

Supplementary Table S3. Raw postoperative opioid consumption data across included studies

Study	Original opioid	Mg, mean ± SD	C, mean ± SD	Dose unit	Data type
**Sousa 2016**	Morphine	5.70 ± 6.20	12.00 ± 6.30	mg	Reported
**Ghaffaripour 2016**	Morphine	0.59 ± 0.04	0.70 ± 0.08	mg/kg	†
**Haryalchi 2017**	Pethidine	10.00 ± 18.92	68.00 ± 17.42	mg	Reported
**Dehkordy 2020**	Morphine	38.00 ± 13.50	53.00 ± 16.00	mg	Reported
**Tsaousi 2020**	Morphine	5.33 ± 3.88	14.58 ± 4.79	mg	Reported
**Sohn 2021**	Fentanyl	284.80 ± 234.70	426.20 ± 268.40	mcg	Reported
**Hadavi 2022**	Morphine	12.50 ± 1.49	19.24 ± 2.81	mg	Reported
**Kamel 2022**	Pethidine	64.20 ± 4.70	71.00 ± 6.20	mg	Reported
**Abdel Rady 2024**	Morphine	18.20 ± 5.50	17.60 ± 5.60	mg	Reported
**Salkaya 2024**	Pethidine	41.00 ± 38.70	38.70 ± 22.70	mg	Reported
**Kim 2025**	Mixed opioids	1.00 ± 3.70	13.00 ± 23.00	IME	Reported
**Tamdogan 2025**	Mixed opioids	35.60 ± 15.20	44.90 ± 14.10	IME	Reported

Abbreviations: Mg, magnesium group; C, control group; IME, intravenous morphine equivalents.
Data are presented as mean ± SD in original units as reported in the included studies.
† Mean and SD were estimated from reported median (IQR).
Opioid doses were subsequently converted to intravenous morphine equivalents (IME) for meta-analysis (see Methods).

Supplementary Table S4. Study-level moderators used in meta-regression analyses

Study	Mean_age_all (years)	Female_prop_all	Loading_dose_mgkg	N_total	Notes
**Sousa 2016**	48.65	1.00	50	36	Bolus + infusion
**Ghaffaripour 2016**	44.08	0.59	30	39	Age from group means
**Ryu 2016**	54.00	0.34	50	74	—
**Haryalchi 2017**	49.50	1.00	0	40	Infusion only
**Dehkordy 2020**	47.80	0.61	50	80	—
**Kim 2021**	64.00	0.00	50	52	Male-only
**Sohn 2021**	56.50	0.54	30	68	—
**Hadavi 2022**	27.39	0.71	30	70	Bolus only
**Shim 2022**	65.38	0.23	30	98	—
**Kanamori 2023**	76.22	0.11	30	63	—
**Kilic 2023**	NR	0.69	50	120	Age NR
**Salkaya 2024**	59.35	0.58	0	60	Infusion only

Values are presented as study-level means or proportions. Female proportion was calculated as the number of female participants divided by total sample size (range 0–1). Loading dose is expressed in $mg{\cdot}kg^{-1}$; studies without a loading dose were coded as 0. NR indicates data not available. Only studies with complete data for the respective moderators were included in meta-regression analyses.

Supplementary Table S5. Results of exploratory random-effects meta-regression analyses

Outcome	Moderator	Type	b	SE	95% CI	p	R^2
**Early pain (0–6 h)**	Mean age	Continuous	0.04	0.02	−0.00 to 0.08	0.07	0.0%
	Female proportion	Continuous	−2.58	0.51	−3.58 to −1.59	<0.001	59.6%
	Magnesium loading dose	Continuous	0.00	0.02	−0.04 to 0.04	0.90	0.0%
	Publication year	Continuous	0.17	0.08	0.02 to 0.32	0.03	26.6%
	Timing (pre-induction + intraoperative vs intraoperative-only)	Binary	−0.02	0.60	−1.20 to 1.16	0.98	0.0%
	Loading dose (>30 vs ≤30 mg·kg⁻¹)	Binary	0.35	0.92	−1.45 to 2.16	0.70	0.0%
	Surgical type (spine vs non-spine)	Binary	0.59	0.48	−0.34 to 1.52	0.21	0.0%
	Trial registration (registered vs non-registered)	Binary	0.11	0.49	−0.85 to 1.07	0.82	2.6%
	Publication period (≤2020 vs >2020)	Binary	1.33	0.35	0.65 to 2.00	<0.001	62.1%
**Pain at 24 h**	Mean age	Continuous	0.02	0.03	−0.05 to 0.08	0.61	0.0%
	Female proportion	Continuous	0.49	0.80	−1.08 to 2.05	0.54	0.0%
	Magnesium loading dose	Continuous	0.02	0.03	−0.05 to 0.08	0.59	0.0%
	Publication year	Continuous	−0.12	0.06	−0.24 to 0.00	0.052	35.7%
	Timing (pre-induction + intraoperative vs intraoperative-only)	Binary	0.16	0.53	−0.89 to 1.20	0.77	0.0%
	Loading dose (>30 vs ≤30 mg·kg⁻¹)	Binary	0.07	0.52	−0.95 to 1.09	0.90	0.0%
	Surgical type (spine vs non-spine)	Binary	−0.70	0.39	−1.47 to 0.07	0.07	25.8%
	Trial registration (registered vs non-registered)	Binary	0.19	0.50	−0.80 to 1.18	0.71	0.0%
	Publication period (≤2020 vs >2020)	Binary	−0.51	0.42	−1.34 to 0.32	0.23	0.0%

Meta-regression analyses were performed using random-effects models. Continuous moderators were analysed using study-level values. Binary moderators were coded as indicated. Female proportion was expressed on a 0–1 scale. Magnesium loading dose is reported in mg·kg⁻¹. R^2 represents the proportion of between-study heterogeneity explained by the moderator.
